# Structural and Histone Binding Ability Characterizations of Human PWWP Domains

**DOI:** 10.1371/journal.pone.0018919

**Published:** 2011-06-20

**Authors:** Hong Wu, Hong Zeng, Robert Lam, Wolfram Tempel, Maria F. Amaya, Chao Xu, Ludmila Dombrovski, Wei Qiu, Yanming Wang, Jinrong Min

**Affiliations:** 1 Structural Genomics Consortium, University of Toronto, Toronto, Ontario, Canada; 2 Department of Biochemistry and Molecular Biology, Center for Gene Regulation, Pennsylvania State University, University Park, Pennsylvania, United States of America; 3 Department of Physiology, University of Toronto, Toronto, Ontario, Canada; University of Oulu, Germany

## Abstract

**Background:**

The PWWP domain was first identified as a structural motif of 100–130 amino acids in the WHSC1 protein and predicted to be a protein-protein interaction domain. It belongs to the Tudor domain ‘Royal Family’, which consists of Tudor, chromodomain, MBT and PWWP domains. While Tudor, chromodomain and MBT domains have long been known to bind methylated histones, PWWP was shown to exhibit histone binding ability only until recently.

**Methodology/Principal Findings:**

The PWWP domain has been shown to be a DNA binding domain, but sequence analysis and previous structural studies show that the PWWP domain exhibits significant similarity to other ‘Royal Family’ members, implying that the PWWP domain has the potential to bind histones. In order to further explore the function of the PWWP domain, we used the protein family approach to determine the crystal structures of the PWWP domains from seven different human proteins. Our fluorescence polarization binding studies show that PWWP domains have weak histone binding ability, which is also confirmed by our NMR titration experiments. Furthermore, we determined the crystal structures of the BRPF1 PWWP domain in complex with H3K36me3, and HDGF2 PWWP domain in complex with H3K79me3 and H4K20me3.

**Conclusions:**

PWWP proteins constitute a new family of methyl lysine histone binders. The PWWP domain consists of three motifs: a canonical β-barrel core, an insertion motif between the second and third β-strands and a C-terminal α-helix bundle. Both the canonical β-barrel core and the insertion motif are directly involved in histone binding. The PWWP domain has been previously shown to be a DNA binding domain. Therefore, the PWWP domain exhibits dual functions: binding both DNA and methyllysine histones.

**Enhanced version:**

**This article can also be viewed as an enhanced version in which the text of the article is integrated with interactive 3D representations and animated transitions. Please note that a web plugin is required to access this enhanced functionality. Instructions for the installation and use of the web plugin are available in Text S1.**

## Introduction

The PWWP domain was first identified as a structural motif of 100–130 amino acids in the WHSC1 (Wolf-Hirschhorn syndrome candidate 1) protein and named after the conserved motif Pro-Trp-Trp-Pro in WHSC1. It was predicted to be a protein-protein interaction domain [1]. Indeed, the PWWP domain of the DNA methyltransferase DNMT3A directly binds SALL3, which functions as an inhibitory factor for DNMT3A. SALL3 expression reduces DNMT3A-mediated CpG island methylation in cell culture and *in vitro* [2]. A mutation in the PWWP domain of DNMT3B diminishes its interaction with the SUMO E3 ligase PIAS1 [3].

The PWWP domain was later on shown to bind DNA in 2002 by Cheng's laboratory [4]. The PWWP domains of the DNA methyltransfearses DNMT3A and DNMT3B are essential for targeting DNA methylation to heterochromatin regions through their chromatin binding ability [5], [6]. HDGF (hepatoma-derived growth factor) and the HRPs (HDGF-related proteins) consist of a highly conserved PWWP domain in their N-terminus and a variable region in the C-terminus. PWWP domains in this subfamily of PWWP-containing proteins also exhibit DNA binding ability and some of these HDGF proteins are implicated in development [7]. HDGF exerts its transcription repressive effect through binding to a conserved DNA element in the promoter region of target genes [8], although it was also reported that it functioned as a nonspecific DNA-binding domain [9]. Another member of this subfamily, PSIP1 (PC4 and SFRS1 interacting protein 1), is a transcriptional coactivator and involved in lentiviral integration. It was shown that the PWWP domain in PSIP1 displays affinity for DNA and chromatin and its chromatin binding ability is crucial for the HIV-1 integration [10], [11]. Recently, PSIP1 was found to promote association of the MLL complex with transcriptionally active chromatin through its PWWP domain [12]. The eukaryotic mismatch repair protein MSH6 also harbors a PWWP domain at its N-terminal region, which binds double-stranded DNA non-specifically [13].

Alongside Tudor, chromodomain, MBT domains, the PWWP domain belongs to the Tudor domain ‘Royal Family’ [14]. The core of the Tudor, MBT and PWWP domains is composed of five β-strands. The canonical chromodomain contains three β-strands that correspond to the middle three β-strands of the Tudor, MBT and PWWP domains, and a C-terminal α-helix. The common function of the ‘Royal Family’ members is their ability to recognize lysine/arginine methylated histones or proteins through an aromatic cage [15], [16], [17]. Although the sequence and structure alignments show that PWWP domains exhibits structural similarity to other ‘Royal Family’ members and most PWWP domains also contain an aromatic cage, it was only recently shown that PWWP is able to bind lysine methylated histone [18], [19], [20], [21].

In order to systematically study the structure and function of this domain, we purified some representative human PWWP domains and tested their binding ability to different histone peptides. The results show that PWWP domain is a weak methyllysine histone binder. Furthermore, we determined the crystal structures of the PWWP domains from seven different human proteins and three PWWP domain complex structures with histone peptides, i.e., BRPF1-H3K36me3, HDGF2-H3K79me3 and HDGF2-H4K20me3. Therefore, the PWWP domain can not only bind DNA but also histones.

## Results and Discussion

### Structures of PWWP domains

**Figure 1:**
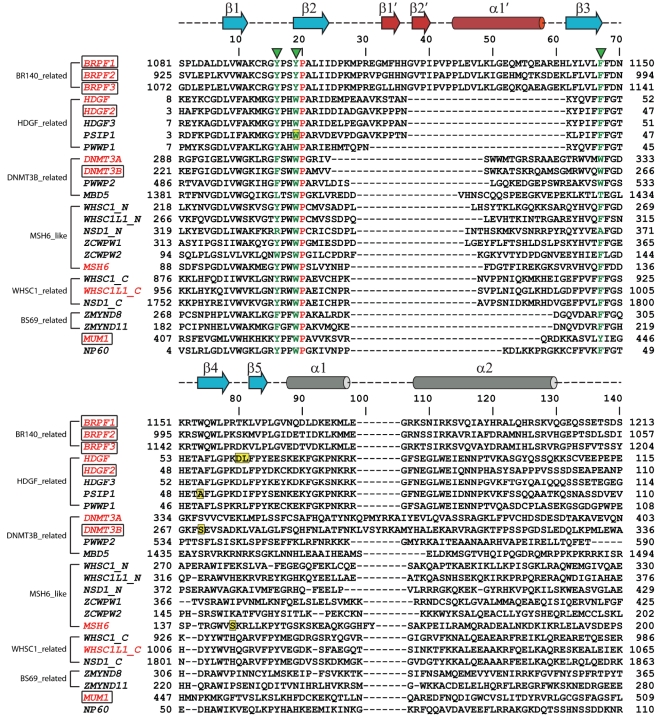
Structure-based sequence alignment of human PWWP domains. There are over 20 PWWP containing proteins in the human genome, which can be categorized into 6 classes. We solved crystal structures of PWWP domains from 7 different human proteins (colored in red and boxed). The structures solved by other labs are colored in red. All of these PWWP domains contain an aromatic cage formed by three aromatic residues (labeled by green triangles) except MBD5 and the N-terminal PWWP domain in NSD1. The identical residues in the alignment are colored in red. The secondary structure elements of BRPF1 are shown on top of the alignment, with cylinders representing helixes and arrows representing strands. The alignment was generated by using Clustal W assisted with manual adjustment.

The PWWP domain was first identified in WHSC1 and named after the central core motif Pro-Trp-Trp-Pro of the PWWP domain in WHSC1 [1]. The PWWP domain comprises 100–130 amine acids and is often present in chromatin-associated proteins. In the human genome, there are at least 22 PWWP domain-containing proteins and three of them contain 2 PWWP domains (WHSC1, WHSC1L1, NSD1). The PWWP domains can be categorized into 6 classes based on sequence homology (Figure 1). The major difference between these different PWWP domains is localized on the insertion motif, which varies in length among the different PWWP domains.

**Figure 2:**
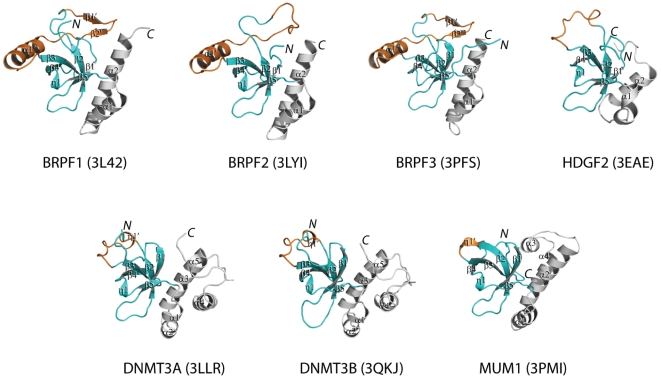
Crystal structures of the seven human PWWP domains reported in this study. PWWP fold consists of three structural elements: the canonical core (colored in cyan) comprising 5 β-strands, which highly resembles the Tudor domain fold, an insertion motif between the second and third β strands (colored in orange) and the PWWP characteristic C-terminal α helix motif consisting of 1–5 α-helixes (colored in grey). The figure was generated by Pymol.

In order to explore the functional roles of the PWWP domains, we determined the crystal structures of the PWWP domains from 7 different human proteins, namely, BRPF1 (bromodomain and PHD finger-containing protein 1), BRPF2, BRPF3, MUM1 (melanoma associated antigen (mutated) 1), DNMT3A, DNMT3B and HDGF2 (Hepatoma-derived growth factor 2). Our structures revealed that the overall fold of the PWWP domain consists of three motifs: a canonical β-barrel core, an insertion motif between the second and third β-strands and a C-terminal α-helix bundle (Figure 2). The canonical β-barrel core harbors an aromatic cage constructed by three aromatic residues, which is a signature feature of the Tudor domain “Royal family” (Figure 1). MBD5 (methyl-CpG binding domain protein 5) and the N-terminal PWWP domain of NSD1 (nuclear receptor binding SET domain protein 1) are two exceptions, which have just one aromatic residue at the conserved positions. A PWWP characteristic C-terminal α helix motif is located in the C-terminal part of the PWWP domains consisting of 1–5 α-helixes. A structure comparison of these PWPP domains shows that the insertion motif between the second and third β strands varies in length and secondary structure among these different classes of PWWP domains. This variable insertion motif is plausibly caused by intron/exon sliding at the genomic level, as the coding region for the second and third β strands are often split by an intron [1].

### Histone binding ability of PWWP domains

The PWWP domain is structurally similar to other members in the Tudor domain ‘Royal family’ [14], and many members in this superfamily have been shown to bind methylated histones [15]. Furthermore, the vast majority of PWWP domains have the aromatic residues in the conserved positions that form a putative methyllysine binding aromatic cage (Figure 1). Therefore, it was compelling to speculate that the PWWP domains may also exhibit methylated histone binding ability, which was proved in recent studies [18], [19], [20], [21]. The *Pombe* protein Pdp1 harbors a PWWP domain in its N-terminus, which was shown to bind mono-methylated histone H4K20. Because the C-terminal fragment of Pdp1 is able to bind the *Pombe* H4K20 methyltransferase SET9, SET9 is recruited to the H4K20me1 chromatin region through the PWWP domain of Pdp1 to increase the concentration of SET9 on chromatin and carry out the trimethylation of histone H4K20 [18]. The PWWP domains of BRPF1 and DNMT3A were reported to bind H3K36me3 [19], [20]. BRPF1 was shown to be present on the actively transcribed gene, and its enrichment corresponds to that of H3K36me3 [20]. DNMT3A was recruited to the chromatin region with the H3K36me3 mark through its interaction of the PWWP domain with H3K36me3 [19].

**Figure 3:**
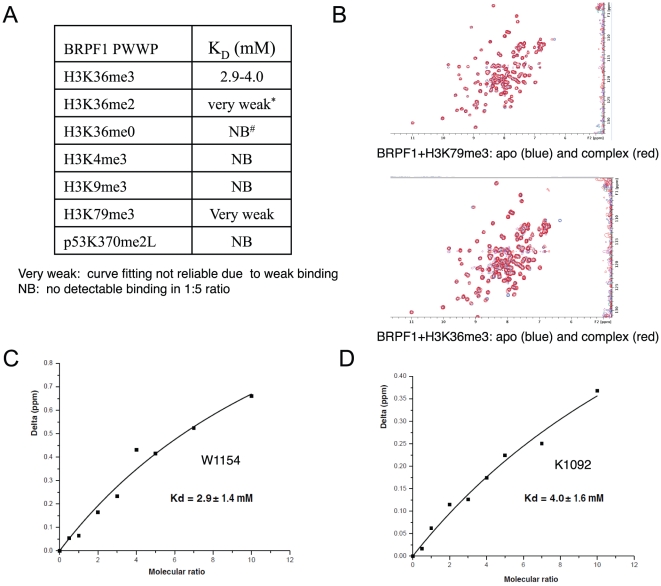
NMR titration confirms that BRPF1 preferentially binds tri-methylated H3K36. (A) Binding affinities of the BRPF1 PWWP domain to different histone peptides. (B)Superposition of ^15^N-^1^H HSQC NMR spectra of the BRPF1 PWWP domain in the presence (red) and absence (blue) of H3K79me3 (1∶10) and H3K36me3 (1∶10) peptides. (C) Binding affinity calculation based on the change in the chemical shifts of W1154 in the ^15^N-^1^H BRPF1 resonances upon addition of nonlabeled H3K36me3 peptide. (D) Binding affinity calculation based on the change in the chemical shifts of K1092 in the ^15^N-^1^H BRPF1 resonances upon addition of nonlabeled H3K36me3 peptide. The concentration of ^15^N BRPF1 in all NMR experiments is 0.2 mM. W1154 and K1092 are two residues from the BRPF1 PWWP domain.

To better understand the histone binding ability and preference of these human PWWP domains, we used fluorescence polarization and NMR titration techniques to measure binding affinities of some representative PWWP domains to various histone peptides bearing different lysine methylation states. By fluorescence polarization assay, we found that the PWWP domains in BRPF1, BRPF2, HDGF2, MUM1 and the N-terminal PWWP domains of WHSC1 and WHSC1L1 show weak binding affinity to histones with H3K36, K3K79 or H4K20 methylation (Table 1). In order to confirm this weak histone binding, we used NMR titration to measure the binding affinity of BRPF1 to different histone peptides. Our NMR titration results show that BRPF1 does not exhibit detectable binding to the H3K4me3 and H3K9me3 peptides, but binds H3K36me3 with a Kd of ∼3 mM (Figure 3), which is consistent with the results reported by Bycroft's group [20]. BRPF1 also shows weaker binding to H3K36me2 and H3K79me3 peptides (Figure 3A). BRPF2 displays a binding preference similar to BRPF1. HDGF2 binds H3K36me3, H3K79me3 and H4K20me3 weakly (Table 1). Consistent with the high throughput binding assay by Mann's group, WHSC1 and WHSC1L1 binds H3K36me3 [21]. It was reported that DNMT3A also binds H3K36me3 [19]. Taken together, similar to other members in the ‘Royal family’, PWWP domain also exhibits methyllysine histone binding ability.

**Table 1:** Binding affinities of human PWWP domains for histone H3 and H4 methylated peptides.

Peptide	Sequence	BRPF1 Kd(mM)	BRPF2 Kd(mM)	HDGF2 Kd(mM)	MUM1 Kd(mM)	WHSC1_N* Kd (mM)	WHSC1L1_N* Kd (mM)
H3K36me2	PATGGVK(me)2KPHRY	0.8±0.2	Very weak	1.0±0.2	0.4±0.1	0.5±0.1	Very weak
H3K36me3	PATGGVK(me)3KPHRY	0.8±0.1	0.9±0.1	Very weak	0.1±0.07	0.6±0.1	Very weak
H3K79me2	EIAQDFK(me)2TDLRY	0.2±0.01	0.2±0.02	Very weak	ND	ND	ND
H3K79me3	EIAQDFK(me)3TDLRY	0.1±0.01	0.4±0.1	Very weak	ND	ND	ND
H4K20me2	AKRHRK(me)2VLRDN	Very weak	ND	Very weak	ND	ND	ND
H4K20me3	AKRHRK(me)3VLRDN	Very weak	ND	Very weak	ND	ND	ND

*: WHSC1 and WHSC1L1 contain two PWWP domains and we only tested the N-terminal PWWP domain.

ND: not determined. Very Weak: the binding is too weak to be fitted reliably.

### Trimethylated lysine histone recognition by the PWWP domains of BRPF1 and HDGF2

To shed light on the molecular mechanism of methylated histone binding by PWWP domains, we determined the crystal structures of the PWWP domain of human BRPF1 in complex with H3K36me3 and that of human HDGF2 in complex with H3K79me3 and H4K20me3.

**Figure 4:**
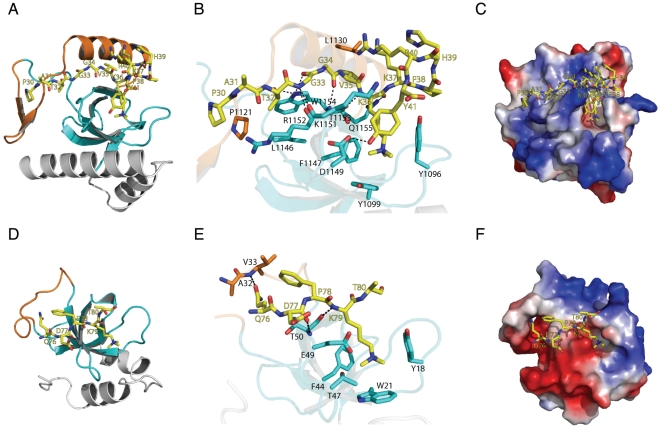
Complex structures of BRPF1-H3K36me3 and HDGF2-H3K79me3. (A) Structure of BRPF1 PWWP domain in complex H3K36me3 peptide. The PWWP domain is shown in cartoon, and the peptide is shown in a stick model. (B) The detailed interactions between the BRPF1 PWWP domain and H3K36me3. Hydrogen bonds are shown in dashed lines. (C) Electrostatic surface representation of BRPF1-H3K36me3 complex. (D) Structure of HDGF2 PWWP domain in complex H3K79me3 peptide. The PWWP domain is shown in cartoon, and the peptide is shown in a stick model. (E) The detailed interactions between the HDGF2 PWWP domain and H3K79me3. Hydrogen bonds are shown in dashed lines. (F) Electrostatic surface representation of HDGF2-H3K79me3 complex.

In the BRPF1-H3K36me3 complex structure, the peptide resides in a groove formed by the insertion motif, the fourth β-strand and its preceding loop from the BRPF1 PWWP domain (Figure 4A–C and Figure S1A). The trimethylated lysine K36 is accommodated in an aromatic cage formed by three aromatic residues (Y1096, Y1099 and F1147). Besides, histone residues H3T32, H3G33 and H3K36me3 make several hydrogen bonds with residues from the fourth β-strand and its preceding loop of BRPF1 PWWP (Figure 4B). Interestingly, the H3Y41 from the histone H3 peptide forms one side of the aromatic cage, but mutating H3Y41 to alanine does not significantly affect the binding of H3K36me3 peptide to BRPF1 (data not shown). We infer that H3Y41 is not involved in the H3K36me3 recognition. Therefore, bothe the canonical β-barrel core and the insertion motif are directly involved in histone binding.

We were also able to co-crystallize HDGF2 with both H3K79me3 and H4K20me3 peptides, which show very similar binding mode. In the HDGF2-H3K79me3 complex structure (Figure 4D–F and Figure S1B–C), the trimethylated lysine K79 is accommodated in an aromatic cage formed by three aromatic residues (Y18, W21 and F44). This trimethylated K79 is the major contributor of the histone binding to the PWWP domain, although histone H3 residues Q76 and D77 also make two hydrogen bonds with V33 from the insertion motif and T50 from the fourth β-strand of the HDGF2 PWWP domain (Figure 4E). This may also explain why HDGF2 shows very weak binding affinity to H3K79me3.

**Figure 5:**
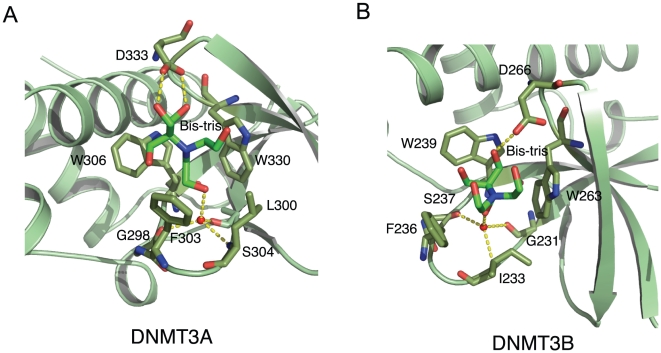
PWWP domains of DNMT3A and DNMT3B bind a bis-tris molecule in their respective aromatic cage. (A) The detailed interactions between DNMT3A and a bis-tris molecule. (B) The detailed interactions between DNMT3B and a bis-tris molecule. The bis-tris molecule is shown in a green stick model, and the interacting residues from the PWWP domain are also shown in stick models. Hydrogen bonds are shown in dashed lines.

**Figure 6:**
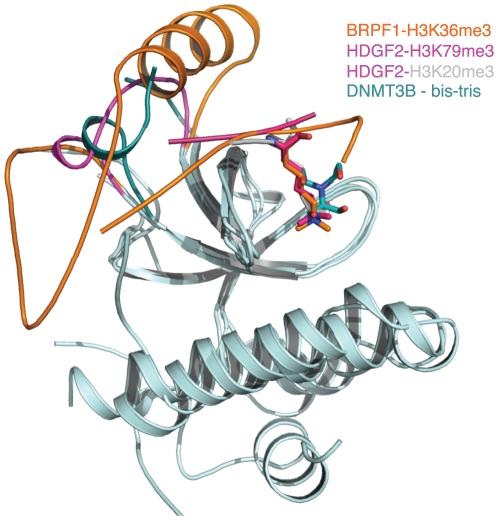
The peptides share a similar binding mode in the complex structures of BRPF1-H3K36me3, HDGF2-H3K79me3 and HDGF2-H4K20me3. The bis-tris molecules in the DNMT3A/DNMT3B complex structures are bound at the same site as the tri-methyl-ammonium group of the methyllysine (DNMT3A structure is not shown here for clarity). The insertion motifs from these three PWWP domains may play a role in conferring the ligand specificity.

DNMT3A had been shown to bind histone H3K36me3 [19], but we were not able to obtain its cocrystals with H3K36me3, Nevertheless, we found a bis-tris buffer molecule in both the DNMT3A and DNMT3B structures (Figure 5 and Figure S1D–E). The propensity of the aromatic cage to bind buffer molecules had been identified before in another ‘Royal family’ member, L3MBTL1 [22], [23]. The bis-tris molecule resides in the conserved aromatic cage of the PWWP domains (Figure 5). Superposition of the DNMT3A and DNMT3B with the BRPF1 and HDGF2 complex structures shows that the bis-tris molecule is bound in the position occupied by the tri-methyl ammonium group of the methyllysine (Figure 6). The bis-tris molecule is bound to DNMT3A and DNMT3B in slightly different conformations. In the DNMT3A structure, the bis-tris molecule forms two hydrogen bonds with the D333 residue from DNMT3A, and three hydrogen bonds with residues G298, L300 and S304 through a conserved water molecule. In the DNMT3B structure, the bis-tris molecule forms one hydrogen bond with the D266 residue from DNMT3B, and three more hydrogen bonds with the conserved residues G231, I233 and S237 via the conserved water molecule. DNMT3A and DNMT3B are DNA methyltransferase, which are essential for de novo methylation and mammalian development [24]. Aberrant DNA methylation is implicated in various diseases, including cancer [25]. The current focus of drug discovery mainly targets on the catalytic domain of DNA methyltransferases. The bis-tris molecule in complex with the PWWP domain of DNMT3A or DNMT3B provides a clue for designing small molecules targeting their histone binding domain.

In all these complex structures, the methyllysine binding aromatic residues are from the loop between the first and second β-strands, the N-terminus of the second β-strand and the C-terminus of the third β-strand (Figure 1). The histone residues C-terminal to the modified lysine do not make significant contributions to the binding (Figure 4), reminiscent of HP1 and Polycomb chromodomains, which mainly binds H3K9me3 and H3K27me3 peptides through residues N-terminal to the respective target lysines [26], [27], [28], [29]. In these complex structures, the insertion motif is directly involved in histone binding, forming one side of the histone binding groove (Figure 6). Furthermore, this insertion motif has different lengths and structures among these PWWP domains (Figure 1 and 6), which may imply that the insertion motif plays a role in determining the ligand specificity.

### Structural comparison of the PWWP domain with the other methyl-lysine binders

**Figure 7:**
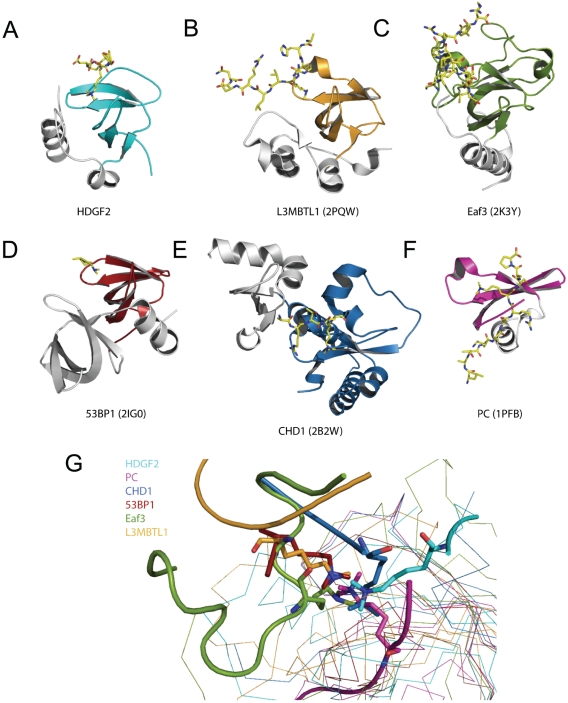
Comparison of the binding modes of methylated histone peptides to PWWP domain, chromo domain, Tudor domain and MBT repeat domain. (A–F) Different histone code reader domains shown individually in complex with their corresponding ligands: HDGF2-H3K36me3 (cyan, A), L3MBTL1-H4K20me2 (yellow, B), Eaf3-a mimic H3K36me2 peptide (green, C), 53BP1-H4K20me2 (red, D), CHD1-H3K4me3 (blue, E), Polycomb-H3K27me3 (magenta, F). The peptides are shown in stick models. (G) Superposition of different histone code “reader” domains in complex with their corresponding ligands. All the structures are shown in the same orientation as they are in Fig. 7A–7F. The conserved β-sheet core among these domains is colored the same as in Fig. 7A–7F.

Comparison of the structural architecture of the PWWP domain to those of chromodomain, MBT and Tudor domains shows that PWWP, MBT and Tudor all have a 5-β-strand canonical core, while the chromodomain consists of three β-strands and one α-helix (Figure 7). Overall, the fold of the PWWP domain has highest structure similarity to that of a single MBT repeat, i.e., the β-strand core is followed by α helixes, which packs against the β barrel core (Figure 7A and 7B). Other domains similar to PWWP are found in Eaf3 and MRG15. Eaf3 and MRG15 bind H3K36me3 through a chromo barrel domain [30], [31], [32]. This chromo barrel domain is structurally similar to the PWWP domain (Figure 7C), but it lacks the PWWP motif, and it harbors a small helix turn between the third and fourth β-strands that lacks in the PWWP domain (Figure 7C). The canonical Tudor domain consists of five β-strands, which can overlay perfectly with the β-barrel core of PWWP (Figure 7D). A typical chromodomain consists of three β-strands and one α-helix, and the three β-strands can be superimposed with the middle three β-strands of PWWP, MBT and Tudor domains (Figure 7E and 7F).

The histone methyllysine binding mode exhibited by PWWP is similar to that adopted by other methyllysine binding proteins [23], [26], [27], [28], [29], [33], [34], [35], [36]. A common feature of these methyllysine binding proteins is that they use an aromatic cage to recognize the methylated lysine [15]. Nevertheless, the histone peptides are bound to their corresponding binders in different orientations (Figure 7G), indicating that the royal family members do not share a common binding cleft, but a similar aromatic cage at an almost identical position. Interestingly, histone peptides bind to a single chromodomain as a β-strand in a position corresponding to the first β-strand of the 5-strand canonical cores of PWWP, Tudor and MBT domains [23], [27], [28], [29], [34], [37], [38] (Figure 7D).

### Mutations in PWWP domain and their implications in functions and diseases

Mutations in PWWP domain-containing proteins have been implicated in different human diseases. The gene *WHSC1* is located in the Wolf–Hirschhorn syndrome critical region on 4p16.3 and is disrupted by chromosomal translocation in lymphoid multiple myeloma disease [39]. It was recently shown that BRPF2 is associated with schizophrenia and bipolar affective disorder [40]. HDGF was reported to be involved in tumorigenesis [41] and the PWWP domain in PSIP1 is critical for chromatin binding and the HIV virus type 1 infectivity [10]. Mutations in MSH6 causes inherited somatic defects in MMR and result in increased development of hereditary non-polyposis colorectal cancer [13]. DNMT3A and DNMT3B are *de novo* DNA methyltransferases and the loss-of-function mutations in human DNMT3B causes a developmental defect characterized by hypomethylation of pericentromeric repeats and are implicated in ICF (immunodeficiency, centromeric instability, facial anomalies) syndrome [24], [42]. So far, the identified point mutations (Figure 1, residues highlighted in yellow) that are implicated in diseases or important for functions are all located either in the aromatic cage or on the fourth β-strand, regions involved in histone and DNA binding.

## Materials and Methods

### Cloning, expression and purification of human PWWP domains

DNA fragment encoding the PWWP domain of human BRPF1 (residues 1085–1213), BRPF2 (residues 925–1049), BRPF3 (residues 1056–1195), HDGF2 (residues 1–93), DNMT3A (residues 278–427), DNMT3B (residues 206–355), MUM1 (residues 406–539) WHSC1 (residues 208–368) and WHSC1L1 (residues 247–402) were amplified by PCR and sub-cloned into pET28-MHL vector (Genbank accession number: EF456735) and transformed into *Escherichia coli* BL21 (DE3)-V2R-pRARE2. The cells were grown in Terrific Broth and the protein was over-expressed by addition of 1 mM isopropyl-1-thio-D-galactopyranoside (IPTG) and incubated overnight at 15°C. Harvested cells were resuspended in 50 mM HEPES, pH 7.4, supplemented with 500 mM NaCl, 2 mM β-mercaptoethanol, 5% glycerol, 0.1% CHAPS. The cells were lysed by passing through a microfluidizer (Microfluidics Corporation) at 20,000 psi. After clarification of the crude extract by high-speed centrifugation, the lysate was loaded onto a 5 ml HiTrap chelating column (GE Healthcare), charged with Ni^2+^. The column was washed with 10 column volumes of 20 mM HEPES buffer, pH 7.4, containing 500 mM NaCl, 50 mM imidazole and 5% glycerol, the protein was eluted with 20 mM HEPES buffer, pH 7.4, 500 mM NaCl, 250 mM imidazole, 5% glycerol. The protein was dialyzed against buffer containing 20 mM HEPES, pH 7.4, 500 mM NaCl and 5% glyceral. TEV protease was added to combined fractions containing target proteins to remove the His-tag. All the proteins except DNMT3A were further purified to homogeneity by ion-exchange chromatography on Source 30S column (10×10) (GE Healthcare), equilibrated with 20 mM PIPES buffer, pH 6.5, and eluted with linear gradient of NaCl up to 500 mM concentration (20CV). For DNMT3A, Source 30Q column was used for ion exchange chromatography. The ^15^N-labeled proteins for NMR titration were purified in the same protocols as native ones except that bacteria were grew in M9 minimal medium containing 1 g/L ^15^(NH_4_)_2_SO_4_ as the sole nitrogen source. The labeled proteins were concentrated to 0.15–0.3 mM for NMR titration.

### Protein crystallization

Purified PWWP domain proteins were crystallized using hanging drop vapor diffusion method at 20°C by mixing 1 µl of the protein solution (10 mg/mL) with 1 µl of the reservoir solution. BRPF1 (apo) and its complex with H3K36me3 peptide were crystallized in 3.5 M sodium formate, 0.1 M Tris-HCl, pH 8.5; BRPF2 in 30% PEG2K-MME, 0.20 M potassium bromide; BRPF3 in 30% PEG 4,000, 0.2 M ammonium sulfate, 0.1 M sodium cacodylate, pH 6.5; HDGF2 in 2.0 M ammonium sulfate, 0.2 M potassium/sodium tartrate, 0.1 M sodium citrate pH 5.6; HDGF2-H3K79me3 complex in 2.0 M ammonium sulfate, 5% isopropanol; MUM1 in 25% PEG 3,350, 0.1 M ammonium sulfate, 0.1 M HEPES, pH 7.5; DNMT3A in 28% PEG 3,350, 0.1 M ammonium sulfate, 0.1 M Bis-Tris, pH 6.0; DNMT3B in 30% PEG2K-MME, 0.20 M potassium bromide, 0.1 M Bis-Tris, pH 6.5. The peptides used for co-crystallization are: SAPATGGVKme3KPHRYR (H3K36me3); EIAQDFK(me)3TDLRY (H3K79me3); AKRHRKme3VLRDN (H4K20me3).

### Fluorescence polarization assay

Fluorescence polarization assays were performed in 384-well plates, using the Synergy 2 microplate reader from BioTek as described in [43]. All the peptides were synthesized and purified by Tufts University Core Services (Boston, MA, U.S.A.), with the N-terminus labeled with fluorescein. Binding assays were performed in a 10 µl volume at a constant labeled peptide concentration (40 nM), by titrating the PWWP domains (at concentrations ranging from low to high micromolar) into 20 mM PIPES buffer (pH 6.5), containing 50 mM NaCl, 0.01% Tween-20. The data points were fitted to ligand binding function using Sigma Plot software to determine the *K*_d_ values.

### NMR

To map the binding site of BRPF1 and HDGF2 PWWP domain for various methylated histone peptides and estimate the corresponding K_d_s, ^15^N-^1^H HSQC spectra were collected with ^15^N-labeled samples of PWWP domains, free and with additions of increasing amounts of unlabeled H3K4me3 (1–11 aa), H3K9me3 (1–15 aa), H3K36me3 (30–41 aa), H3K79me3 (73–84 aa), p53K370me2 (365–375 aa), p53K372me2 (364–376 aa) and p53K382me2 (376–388 aa) peptides. Weighted average chemical shift variations (Δ ppm) were calculated according to the formula (Δ ppm = ([δH^N^]^2^+[δN]^2^)^½^, where δH^N^ and δN are the changes in HN and N chemical shifts, respectively) as described in [30]. From the Δppm, the K_d_s were estimated with the amide peaks of two selected amino acids, as shown in Figure 4. The shifted BRPF1 resonances are assigned according to the recent publication [20].

### Data Collection and Structure Determination

All diffraction data were collected at 100 K and reduced with the HKL suite of programs [44]. To obtain phase information for BRPF1, 436 0.5° oscillation images collected on an FR-E copper rotating anode source (Rigaku) on a selenomethionyl derivative [45] crystal of space group I222 (a = 43.3 Å, b = 72.0 Å, c = 114.0 Å). The structure was solved with the single wavelength anomalous diffraction (SAD) method [46] using the programs SHELXD and SHELXE [47]. An initial model was build automatically with the program ARP/wARP [48]. The model was further refined against a dataset that was derived from 406 0.5° oscillation images collected at beamline 19ID of the Advanced Photon Source at a wavelength of 0.977 Å. COOT [49], REFMAC [50], and MOLPROBITY [51] were used for interactive model building, refinement and validation, respectively. The crystal structures of DNMT3A, DNMT3B, BRPF2, BRPF3, MUM1 and the complex structures of BRPF1-H3K36me3, HDGF2-H3K79me3 and HDGF2-H4K20me3 were solved by molecular replacement using MOLREP [52], and refined using a similar protocol to that of apo-BRPF1. Crystal diffraction data and refinement statistics are displayed in Tables 2 and 3.

**Table 2:** Crystallography data and refinement statistics.

	BRPF1	BRPF1+H3K36me3	BRPF2	BRPF3	HDGF2+H4K20me3
**PDB Code**	3L42	3MO8	3LYI	3PFS	3QBY
**Data collection**					
Space group	P4_3_2_1_2	I222	P2_1_2_1_2_1_	P2_1_2_1_2	P2_1_2_1_2_1_
Cell dimensions					
*a*, *b*, *c* (Å)	44.35, 44.35, 122.72	43.09, 70.61, 113.54	45.03, 46.08, 130.71	68.41,134.52,30.23	41.53, 41.68, 156.75
*α*, *β*,*γ* (°)	90, 90, 90	90, 90, 90	90, 90, 90	90, 90, 90	90, 90, 90
Resolution (Å) (highest resolution shell)	40.00-1.30 (1.32-1.30)	50.0 – 1.70 (1.74-1.70)	50.0 – 2.10 (2.18 – 2.10)	50.00-1.90 (1.97-1.90)	25.00-1.94 (1.97-1.94)
Unique reflections	31403	19545	16475	22879	21026
*R*_merge_	5.6 (46.5)	6.2 (40.7)	7.4 (49.5)	9.1 (47.6)	3.9 (18.2)
*<I>/<σ(I)>*	65.7 (7.9)	30.0 (3.5)	24.6 (3.2)	9.5(4.6)	58.7 (9.2)
Completeness (%)	99.9 (100.0)	99.4 (96.7)	99.0 (95.3)	99.9 (100.0)	99.9 (98.3)
Redundancy	14.5 (13.8)	7.7 (6.0)	6.7 (5.0)	6.0 (6.0)	6.4 (5.4)
**Refinement**					
Resolution (Å)	30.00-1.30	60.0-1.70	37.7 – 2.10	67.3-1.90	20.00-1.95
No. reflections (of which test set)	31089 (1565)	18540 (1005)	15594 (827)	21660(1170)	20561 (1055)
*R*_work/_ *R*_free_ (%)	20.9/23.3	19.2/20.7	23.4/27.6	21.3/25.9	21.6/25.3
No. atoms					
Protein	1056	1041	1802	2099	2021
Ligand	0	96	0	0	22
Water	109	107	78	136	52
B-factors (Å^2^)					
Protein	13.1	20.1	43.4	17.0	34.5
Ligand	N/A	22.7	N/A	N/A	53.9
Water	20.2	28.8	46.7	25.2	36.9
R.m.s deviations					
Bond lengths (Å)	0.016	0.011	0.012	0.011	0.011
Bond angles (°)	1.63	1.21	1.36	1.20	1.21
Ramachandran plot % residues					
Favored	95.3	94.6	93.7	98.1	93.7
Additionally allowed	4.7	5.4	6.3	1.9	6.3
Generously allowed	0.0	0.0	0.0	0.0	0.0
Disallowed	0.0	0.0	0.0	0.0	0.0

**Table 3:** Crystallography data and refinement statistics.

	HGDF2	HDGF2+H3K79me3	MUM1	DNMT3A	DNMT3B
**PDB Code**	3EAE	3QJ6	3PMI	3LLR	3QKJ
**Data collection**					
Space group	P2_1_2_1_2_1_	P6_1_	P2_1_2_1_2_1_	P2_1_	P3_2_
Cell dimensions					
*a*, *b*, *c* (Å)	41.56, 59.95, 104.94	74.93, 74.93, 41.76	55.04, 57.72, 187.07	67.11, 83.99, 74.59	74.54, 74.54 160.27
*α*, *β*,*γ* (°)	90, 90, 90	90, 90, 120	90, 90, 90	90, 90.48, 90	90, 90, 120
Resolution (Å) (highest resolution shell)	40.00-2.25 (2.33-2.25)	40.00-2.30 (2.34-2.30)	50.0-2.85 (2.95-2.85)	50.00-2.30 (2.40-2.30)	50.0 – 2.05 (2.11 – 2.04)
Unique reflections	12838	6094	14914	36557	62131
*R*_merge_	8.2 (56.5)	8.5 (46.6)	0.110 (52.2)	10.9(34.6)	7.1 (60.6)
*<I>/<σ(I)>*	24.4 (2.3)	43.9 (6.2)	17.5 (3.9)	12.4(4.2)	12.3 (2.0)
Completeness (%)	96.7 (83.5)	100.0 (100.0)	99.9 (100.0)	99.0(98.5)	98.9 (97.8)
Redundancy	6.4 (4.6)	10.8 (10.4)	6.8 (7.1)	3.8(3.8)	3.6 (3.4)
**Refinement**					
Resolution (Å)	40.00-2.24	37.47-2.30	50.0-2.82	50.10-2.30	34.04-2.04
No. reflections (of which test set)	12148 (632)	6063 (281)	14110 (751)	34711 (1819)	59117 (3163)
*R*_work/_ *R*_free_ (%)	22.4/26.0	23.4/27.6	22.8/30.4	22.4/25.9	22.6/27.1
No. atoms					
Protein	1322	719	3797	5373	4200
Ligand	0	45	0	70	56
Water	45	10	0	362	321
B-factors (Å^2^)					
Protein	53.1	42.0	18.5	15.4	49.6
Ligand	N/A	46.7	N/A	26.9	57.4
Water	51.0	32.3	N/A	20.9	56.4
R.m.s deviations					
Bond lengths (Å)	0.012	0.011	0.011	0.006	0.023
Bond angles (°)	1.26	1.17	1.29	0.95	1.90
Ramachandran plot % residues					
Favored	95.3	91.5	90.3	96.9	95.7
Additionally allowed	4.7	8.5	9.0	1.5	3.2
Generously allowed	0.0	0.0	0.7	1.6	1.2
Disallowed	0.0	0.0	0.0	0.0	0.0

*: Values in parentheses correspond to the highest resolution shells.

# *R_merge_* = Σ*_hkl_*Σ*_j_*∣*I*(*hkl;j*)−<*I*(*hkl*)>|/(Σ*_hkl_*Σ*_j_*<*I*(*hkl*)>), where *I*(*hkl;j*) is the *j*th measurement of the intensity of the unique reflection (*hkl*), and *I*(*hkl*) is the mean overall symmetry related measurements.

§RMSD: root mean squared deviation.

## Supporting Information

Figure S1 **Electron density maps for the ligands identified in our complex structures and reported in this paper.** (A) The omit density map for the H3K36me3 peptide in the BRPF1-H3K36me3 complex at 3σ contour. (B) The omit density map for the H3K79me3 peptide in HDGF2+H3K79me3 at 3σ contour. (C) The omit density map for the H4K20me3 peptide in HDGF2+H4K20me3 at 2σ contour. (D) the 2Fo-Fc density map for the bis-tris molecule in the DNMT3A-bis-tris structure. (E) the 2Fo-Fc density map for the bis-tris molecule in the DNMT3B-bis-tris structure.
(EPS)

Datapack S1 **Standalone iSee datapack - contains the enhanced version of this article for use offline.** This file can be opened using free software available for download at http://www.molsoft.com/icm_browser.html.
(ICB)

Text S1 **Instructions for installation and use of the required web plugin (to access the online enhanced version of this article).**
(PDF)
